# Surface Coordination‐Dependent Oxygen Evolution Pathways in Rutile IrO_2_

**DOI:** 10.1002/advs.78589

**Published:** 2026-11-05

**Authors:** Gahee Noh, Seong‐Sik Kim, Hyungwoo Lee, Jaejin Hwang, Michael Patton, Owen Ericksen, Tae Gyu Yun, Seung‐Hyun Heo, Suho Lee, Yurim Hong, In Su Lee, Daesu Lee, Chang‐Beom Eom, Jaekwang Lee, Minseok Choi, Sung‐Yoon Chung, Si‐Young Choi

**Affiliations:** ^1^ Department of Materials Science and Engineering Pohang University of Science and Technology (POSTECH) Pohang Republic of Korea; ^2^ Department of Physics Inha University Incheon Republic of Korea; ^3^ Department of Physics Pusan National University Busan Republic of Korea; ^4^ Department of Materials Science and Engineering University of Wisconsin‐Madison Madison USA; ^5^ Department of Materials Science and Engineering Korea Advanced Institute of Science and Technology (KAIST) Daejeon Republic of Korea; ^6^ Department of Physics Pohang University of Science and Technology (POSTECH) Pohang Republic of Korea; ^7^ Department of Chemistry Pohang University of Science and Technology (POSTECH) Pohang Republic of Korea; ^8^ Physics Research Institute Inha University Incheon Republic of Korea; ^9^ Center for Van der Waals Quantum Solids Institute for Basic Science (IBS) Pohang Republic of Korea; ^10^ Department of Semiconductor Engineering Pohang University of Science and Technology (POSTECH) Pohang Republic of Korea; ^11^ Analysis Science & Engineering Team Samsung Electronics Hwaseong Republic of Korea

**Keywords:** catalysis, crystal orientation, electronic structure, iridium oxide, oxygen evolution, scanning transmission electron microscope

## Abstract

Rutile iridium dioxide (IrO_2_) is renowned as an effective catalyst for the oxygen evolution reaction (OER), yet how its atomic structure governs orientation‐dependent activity remains unclear. Here, we investigate epitaxial rutile IrO_2_ thin films with crystallographically well‐defined (110), (101), and (001) orientations and show that their OER activity follows the order: (110) > (101) > (001). Atomic‐resolution scanning transmission electron microscopy (STEM) analysis identifies distinct surface terminations and Ir‐O coordination motifs that differ from commonly assumed idealized surface models. Experimentally informed electronic‐structure calculations reveal that the (110) and (101) surfaces exhibit enhanced O 2*p* states near the Fermi level, favoring oxygen‐involved charge transfer, whereas the (001) surface shows a more Ir‐centered electronic character. Reaction‐barrier calculations further indicate an energetic preference for the quadruple oxygen coupling mechanism (QOCM) on the (110) and (101) surfaces, whereas the (001) surface is more consistent with an Ir‐centered adsorbate evolution mechanism (AEM)‐like pathway. These results establish surface coordination as a key descriptor linking atomic termination, electronic structure, reaction pathway, and OER activity, providing a basis for designing high‐performance IrO_2_ catalysts through surface‐structure engineering.

## Graphical abstract

Epitaxial rutile IrO_2_ surfaces with crystallographically distinct orientations reveal a strong dependence of oxygen evolution reactivity on surface atomic coordination. The (110) and (101) surfaces exhibit O‐rich electronic character and an energetic preference for the quadruple oxygen coupling mechanism (QOCM), whereas the (001) surface shows more Ir‐centered character and favors an adsorbate evolution mechanism (AEM)‐like pathway.

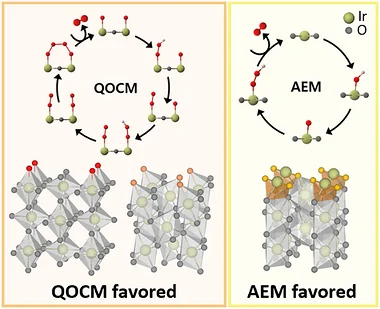

## 1 Introduction

The oxygen evolution reaction (OER) is essential for sustainable hydrogen production through water electrolysis [1, 2, 3, 4, 5]. However, the sluggish kinetics of the multi‐electron OER process remain a major bottleneck limiting the overall efficiency of electrochemical energy conversion systems. Rutile iridium dioxide (IrO_2_) has been widely recognized as one of the most effective OER catalysts, particularly under acidic conditions, owing to its exceptional catalytic activity and superior stability [6, 7]. This performance is generally attributed to its favorable adsorption energetics and optimal electronic structure that lowers the OER overpotential [7, 8].

Despite extensive studies on IrO_2_, the fundamental origin of its outstanding catalytic activity remains actively debated, particularly regarding the nature of the active sites and the dominant reaction pathways. Recent studies have suggested that the OER activity of rutile IrO_2_ strongly depends on crystallographic surface orientation because of the intrinsic structural anisotropy of the rutile lattice [9, 10]. Different surfaces expose distinct atomic configurations, coordination environments, and surface bonding states, which can significantly influence adsorption energetics and catalytic reaction pathways [11].

Catalytic reactions are inherently surface‐mediated processes. The precise surface termination and local coordination environment play a decisive role in determining the reaction mechanism [11]. In rutile IrO_2_, the connectivity of IrO_6_ octahedra gives rise to various surface atomic configurations with different Ir‐O coordination environments, including 4‐, 5‐, and 6‐fold coordinated surface Ir atoms. These distinct local coordination motifs can drastically alter the adsorption sites of OER intermediates and thereby determine whether the catalytic pathway proceeds through an Ir‐centered or oxygen‐involving mechanism. The conventional OER mechanism, the adsorbate evolution mechanism (AEM), assumes that Ir atoms act as the primary adsorption sites for oxygen intermediates [10]. In contrast, more recent studies have also proposed alternative pathways, including the oxide path mechanism (OPM), which involves direct O–O coupling between oxygenated species adsorbed on neighboring metal sites, as well as oxygen‐involving pathways such as the lattice oxygen mechanism (LOM) and the quadruple oxygen coupling mechanism (QOCM), in which surface oxygen atoms directly participate in O_2_ formation. These oxygen‐involving mechanisms have attracted significant attention because they may explain the high activity of IrO_2_ beyond the conventional AEM. Although the LOM can enhance catalytic activity, it is often accompanied by lattice oxygen exchange and structural degradation. In contrast, the QOCM may provide a more stable oxygen‐coupling pathway by promoting O─O bond formation without substantial disruption of the Ir–O framework [12, 13, 14, 15]. Previous studies on OER mechanisms have often assumed idealized surface coordination structures based primarily on computational models, without direct experimental identification of the actual surface terminations [10, 11, 12, 13, 14, 15]. Consequently, establishing a direct correlation between experimentally identified surface coordination and OER mechanisms is crucial to move beyond idealized assumptions and clarify the true catalytic pathways operating on IrO_2_.

Here, we investigate the surface coordination‐governed OER activity of epitaxial IrO_2_ thin films and establish a direct correlation between surface atomic structure, electronic structure, and catalytic behavior [16, 17, 18, 19, 20]. Our study provides experimental evidence that the OER activity and mechanism of IrO_2_ are critically governed by surface orientation and the corresponding surface electronic structure. These findings establish a fundamental framework for understanding orientation‐dependent catalytic mechanisms and provide a rational strategy for designing high‐performance IrO_2_‐based electrocatalysts through surface‐structure engineering.

## 2 Results and Discussion

### 2.1 Synthesis and Structural Characterization

To compare intrinsic orientation‐dependent OER activity, we first prepared atomically flat and crystallographically well‐defined IrO_2_ thin films. X‐ray diffraction (XRD) was used to confirm epitaxial growth and out‐of‐plane orientation, and atomic force microscopy (AFM) verified the atomic‐level flatness of the films. We synthesized high‐quality IrO_2_ films with (110), (101), and (001) orientations on rutile TiO_2_ substrates via radio‐frequency (RF) magnetron sputtering (details are provided in the Experimental Section). As shown in Figure S1, IrO_2_ films and TiO_2_ substrates share the same rutile crystal structure, with the lattice parameter *c* shorter than *a* and *b* (IrO_2_; *a* and *b* = 4.51 Å, *c* = 3.16 Å, TiO_2_; *a* and *b* = 4.59 Å, *c* = 2.95 Å). The XRD θ–2θ scans in the top panels of Figure 1a–c demonstrate that IrO_2_ films grow epitaxially on TiO_2_ substrates with (110), (101), and (001) orientations. The AFM images in the bottom panels of Figure 1a–c and Figure S2 further reveal that all three films exhibit remarkably low surface roughness across multiple regions. The corresponding root‐mean‐square (RMS) values, summarized in Table S1, are below 0.5 nm. This level of smoothness is significantly lower than that reported for previously studied IrO_2_ thin films [10], suggesting that the intended orientation‐controlled growth was successfully achieved.

**FIGURE 1:**
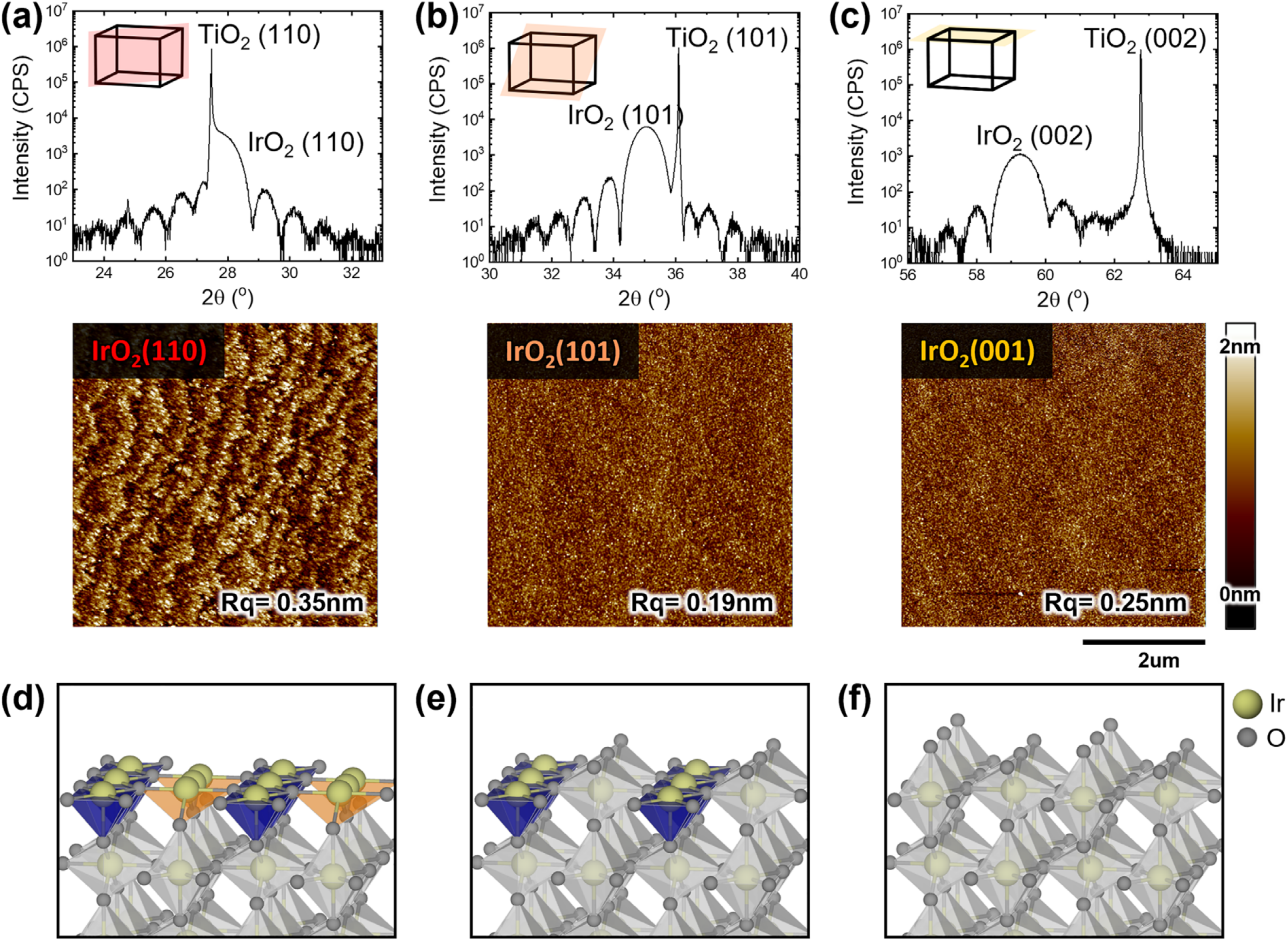
Epitaxial IrO_2_ thin films and coordination‐dependent surface termination models. (a–c) XRD 𝜃‐2𝜃 scans and atomic force microscopy (AFM) for IrO_2_ films on a TiO_2_ substrates with (110), (101), and (001) orientations, respectively. The XRD results verify the epitaxial growth of the films, and the AFM images reveal atomically flat surfaces with low RMS roughness (Rq < 0.5 nm). (d–f) Surface coordination candidates of the IrO_2_ (110) surface. (d) All surface Ir atoms can function as adsorption sites, (e) only the 5‐fold coordinated surface Ir atoms can serve as adsorption sites, (f) surface Ir atoms no longer act as adsorption sites, and surface oxygen atoms instead serve as the adsorption sites.

OER intermediates interact first with exposed surface sites; thus, the local Ir‐O coordination environment can strongly influence the reaction pathway [21, 22, 23]. In IrO_2,_ the IrO_6_ octahedra are connected through corner‐sharing and edge‐sharing. The surface termination can change the Ir‐O coordination environment, causing IrO_6_ coordination to IrO_5_ (square‐pyramidal) or IrO_4_ (distorted tetrahedral) coordination. (Figure S3) Since OER intermediates such as ^*^OH, ^*^O, and ^*^OOH bind to the surface through oxygen atoms, electrophilic and coordinatively unsaturated Ir sites are likely to serve as the preferred adsorption sites [24]. Therefore, surface Ir‐O polyhedra exposing undercoordinated Ir atoms, such as IrO_4_ or IrO_5_ motifs, are expected to promote an Ir‐centered AEM. Meanwhile, surfaces dominated by fully coordinated IrO_6_ motifs lack accessible undersaturated Ir sites, making direct adsorption of oxygen intermediates on Ir less favorable. In this case, surface oxygen atoms may instead act as the primary adsorption sites, and Ir participates in the reaction indirectly by stabilizing the adsorption intermediates [14].

Based on this coordination‐site criterion, Figure 1d–f illustrates the feasible surface terminations of IrO_2_ (110) with different Ir‐O coordination environments. Figure 1d shows a highly undercoordinated termination composed of IrO_4_ and IrO_5_ motifs, whereas Figure 1e represents the stoichiometric IrO_2_ (110) surface model composed of IrO_5_ and IrO_6_ [25, 26, 27]. Because both structures retain accessible undercoordinated Ir sites, they are expected to favor AEM [28]. In contrast, Figure 1f depicts a termination where surface Ir atoms are in IrO_6_ coordination. This configuration suppresses direct Ir‐site adsorption and is therefore more consistent with an oxygen‐centered pathway like QOCM, rather than the AEM [14, 15]. These models suggest that the surface Ir‐O coordination environment can govern the nature of the active sites and thereby alter the preferred OER pathway. Therefore, precise identification of the actual surface termination is essential for correlating the surface atomic structure with the dominant reaction mechanism.

### 2.2 Orientation‐Dependent Structural Properties

Scanning transmission electron microscopy (STEM) imaging is a powerful technique for directly visualizing the surface atomic structures, sometimes revealing how surface reconstruction forms [29, 30, 31]. To identify the terminations of IrO_2_ (110), (101), and (001), we performed atomic‐resolution high‐angle annular dark‐field (HAADF)‐STEM and enhanced annular bright‐field (eABF)‐STEM analysis. HAADF‐STEM imaging provides strong Z‐contrast, enabling clear visualization of heavy atoms like iridium (Figures S4 and S5). However, its sensitivity to light elements such as oxygen is limited. Therefore, eABF‐STEM was employed to complement HAADF‐STEM by enhancing oxygen contrast near the surface (details are provided in the Experimental Section).

Figure 2a–c present HAADF images showing the overall structural quality of (110), (101), and (001) films, respectively. The corresponding indexed fast Fourier transform (FFT) patterns exhibit orientation‐specific reflections, further confirming the distinct crystallographic orientations of the three films (Figure S6). All three films exhibit atomically flat and well‐oriented surfaces without noticeable defects. The corresponding high‐magnification HAADF‐STEM images in the left panels of Figure 2d–f further resolve the Ir framework at the surface region. In all orientations, the Ir columns remain continuous from the interior lattice to the outermost surface, providing a reliable crystallographic reference for assigning the surface terminations. In the right panels of Figure 2d–f, eABF‐STEM images reveal oxygen‐containing surface terminations of IrO_2_ films. For the IrO_2_ (110) plane, images acquired along two projections show that all surface Ir atoms retain IrO_6_ coordination (Figure 2d and Figure S7a), in contrast to conventional models that assume half of the IrO_5_ state [25, 26, 27]. Similarly, the IrO_2_ (101) plane also exhibits IrO_6_ coordination (Figure 2e and Figure S7b), deviating from prior reports that described the surface as having Ir atoms in IrO_5_ [25]. The absence of coordinatively unsaturated Ir sites on these planes suggests that AEM may be less favorable, whereas an oxygen‐involving pathway may become more relevant. In contrast to the (110) and (101) surfaces, the eABF‐STEM images of the IrO_2_ (001) surface exhibit a less uniform surface coordination, with slight distortions in the Ir‐O octahedra within the top 1–2 atomic layers (Figure 2f), consistent with its higher surface energy [25]. The predominance of IrO_4_ coordination increases the density of coordinatively unsaturated Ir sites, thereby favoring the Ir‐centered pathway.

**FIGURE 2:**
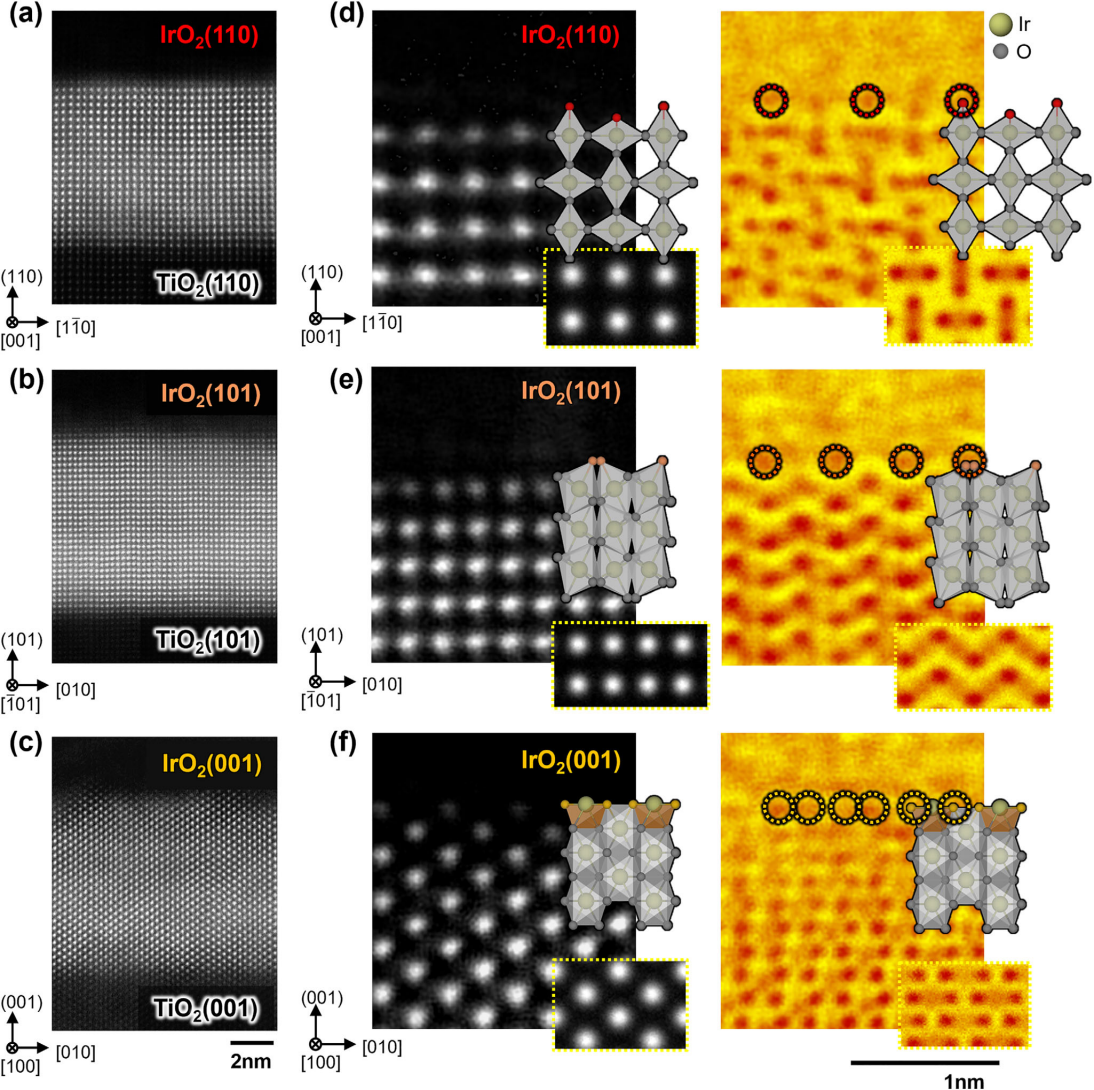
Direct observation of minimally reconstructed structures of IrO_2_ (110), (101), and (001) surfaces. (a–c) Low‐magnification HAADF‐STEM images of IrO_2_ (110), (101), and (001) surfaces, respectively, demonstrating atomically flat surfaces across all IrO_2_ thin films. (d–f) High‐magnification HAADF‐STEM (left) and eABF‐STEM (right) images. The yellow dashed boxes indicate the simulated HAADF and eABF images of IrO_2_. The overlaid atomic models compare the projected bulk framework with the experimentally identified terminations. HAADF‐STEM highlights the Ir framework, whereas eABF‐STEM provides complementary oxygen contrast.

Notably, despite their distinct surface terminations, HAADF‐ and eABF‐STEM images in Figure 2d–f show that all three surfaces maintain largely bulk‐like atomic frameworks. This observation is in line with previous findings on rutile IrO_2_, which proposed that the bulk‐like structure can be largely preserved with only minor surface relaxation [32]. This behavior can be associated with the robust Ir^4+^–O framework and metallic character of IrO_2_, which may relieve surface instability through slight electronic redistribution and local relaxation [33, 34]. However, the underlying mechanism responsible for the limited reconstruction is not yet fully established, and additional experimental and theoretical investigations are needed. These observations establish the actual surface coordination environment of IrO_2_, providing a structural basis for understanding orientation‐dependent OER behavior. Nevertheless, these coordination‐based predictions must be electronically examined because the operative pathway depends not only on site geometry but also on charge‐transfer accessibility of Ir and oxygen. Therefore, we analyze surface electronic structure near the Fermi level for the experimentally identified surface coordination.

As shown in Figure S8, the bulk IrO_2_ electronic structure reveals a high density of the Ir 5*d* band near the Fermi level, whereas the O 2*p* band exhibits a significantly lower density, indicating that iridium can readily participate in OER rather than oxygen. On the other hand, the projected density of states (DOS) of IrO_2_ surfaces in Figure 3a–c reveals that the (110) and (101) surfaces possess enhanced O 2*p* states near the Fermi level, indicating favorable oxygen‐involved charge transfer. In contrast, the (001) surface exhibits Ir 5*d* states positioned closer to the Fermi level than the O 2*p* states, suggesting a reduced contribution of surface oxygen and a more Ir‐centered electronic character. To quantitatively evaluate the relative tendency toward oxygen‐involved OER pathways, we employed band‐center analysis, as summarized in Figure 3d [12, 35].

**FIGURE 3:**
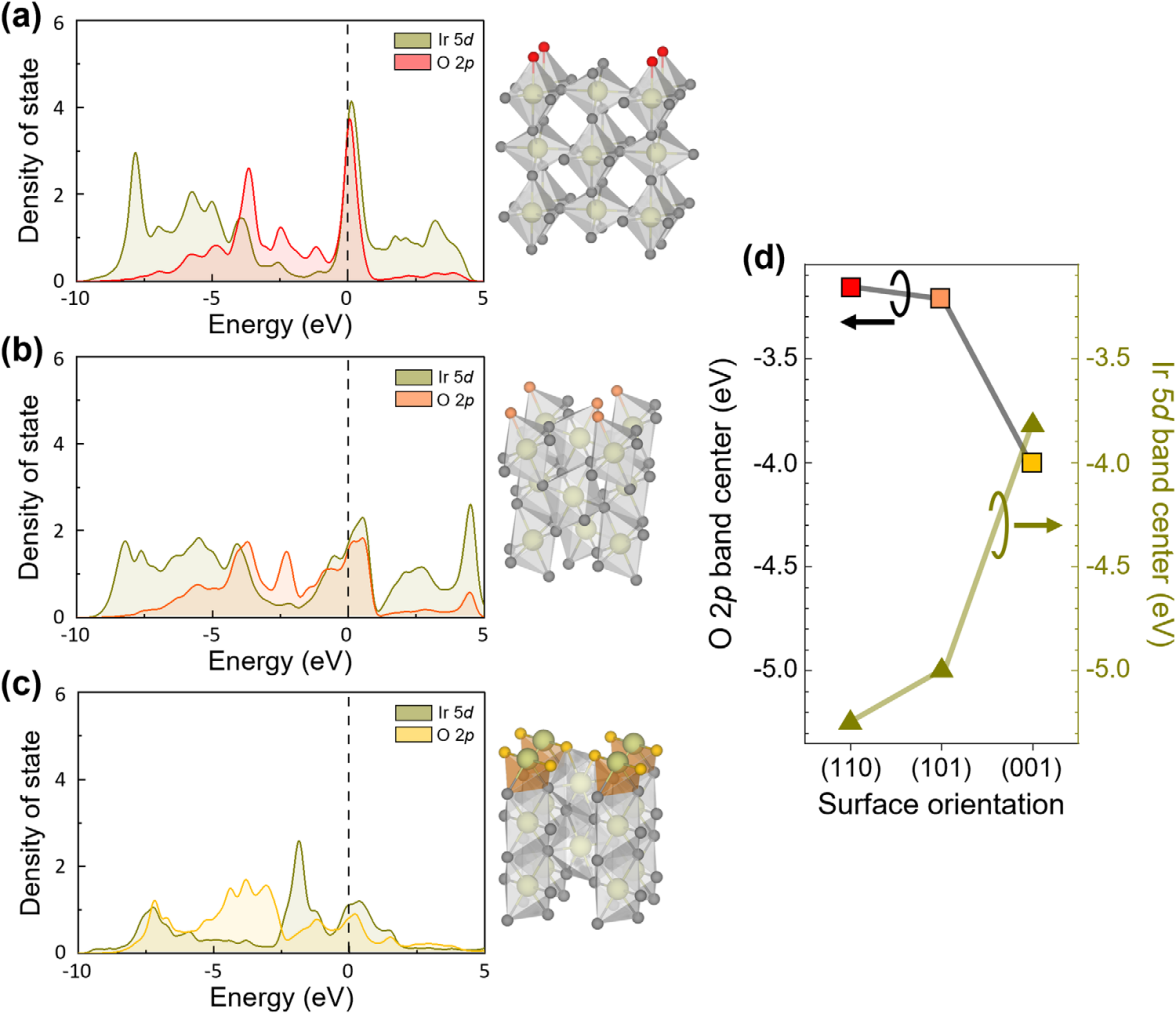
Electronic Structures of IrO_2_ (110), (101), and (001) Surfaces. (a–c) Surface‐projected DOS of IrO_2_ (110), (101), and (001), calculated using the experimentally identified surface atoms shown in the corresponding structural models. (a) For the IrO_2_ (110) surface, the electronic structure of the surface oxygen atoms (highlighted in red) and their bonded iridium atoms. (b) For the IrO_2_ (101) surface, the electronic structure of the surface oxygen atoms (highlighted in blue) and their bonded iridium atoms. (c) For the IrO_2_ (001) surface, the electronic structure of the surface oxygen atoms (highlighted in green) and their bonded iridium atoms. (d) Comparison of the O 2*p* and Ir 5*d* band centers for each surface coordination.

The (110) surface exhibits the O 2*p* band center closest to the Fermi level, followed by the (101) surface, suggesting that oxygen‐involving electron transfer is most favorable on the (110) surface and less pronounced on the (101) surface. By contrast, the (001) surface shows an Ir 5*d* band center located closer to the Fermi level than the O 2*p* band center, reflecting a stronger Ir‐centered electronic character. Importantly, the relaxed slab structures show only minor relaxation, consistent with Figure 2 and Figure S9, supporting the use of these experimentally informed models for electronic‐structure analysis. Taken together, these electronic descriptors suggest a qualitative activity trend of (110) > (101) > (001), because enhanced oxygen participation in catalysis may enable oxygen‐involved charge transfer beyond the conventional Ir‐centered AEM.

### 2.3 Orientation‐Dependent Catalytic Mechanism

To determine whether the surface‐dependent electronic tendency inferred from DOS translates into distinct OER pathways, we compared the reaction energetics of the Ir‐centered AEM and the oxygen‐centered QOCM and additionally evaluated the OPM as a competing dual‐site pathway. This comparison is motivated by our STEM results, which reveal that the (110) and (101) surfaces are dominated by fully coordinated IrO_6_ motifs, whereas the (001) surface exposes undercoordinated Ir sites with an IrO_4_ coordination environment. The LOM was not included because lattice‐oxygen participation has been associated with Ir‐vacancy‐mediated activation, whereas our STEM observations reveal largely bulk‐truncated surfaces without obvious Ir‐vacancy‐rich reconstruction. For the (110) and (101) surfaces, the examined OPM pathway exhibits substantially higher activation barriers of 1.70 and 1.83 eV, respectively, than the characteristic QOCM steps, indicating that OPM is kinetically less favorable for the experimentally identified surface structures (Figure S10). For the (001) surface, an analogous OPM pathway was not considered because the experimentally identified termination does not provide a geometrically suitable neighboring Ir‐site configuration for the required O–O coupling. Figure 4a illustrates a schematic of the AEM, in which the reaction proceeds through sequential adsorption and transformation of oxygen intermediates such as ^*^OH, ^*^O, and ^*^OOH on the metal site, followed by O_2_ release [28]. In contrast, the QOCM involves the direct participation of surface oxygen atoms in OER, rather than acting merely as spectator species [14, 15]. As depicted in Figure 4b, QOCM involves the coupling of multiple surface oxygen atoms to form O_2_, while Ir plays an indirect role in facilitating the coupling of four oxygen atoms to form O_2_.

**FIGURE 4:**
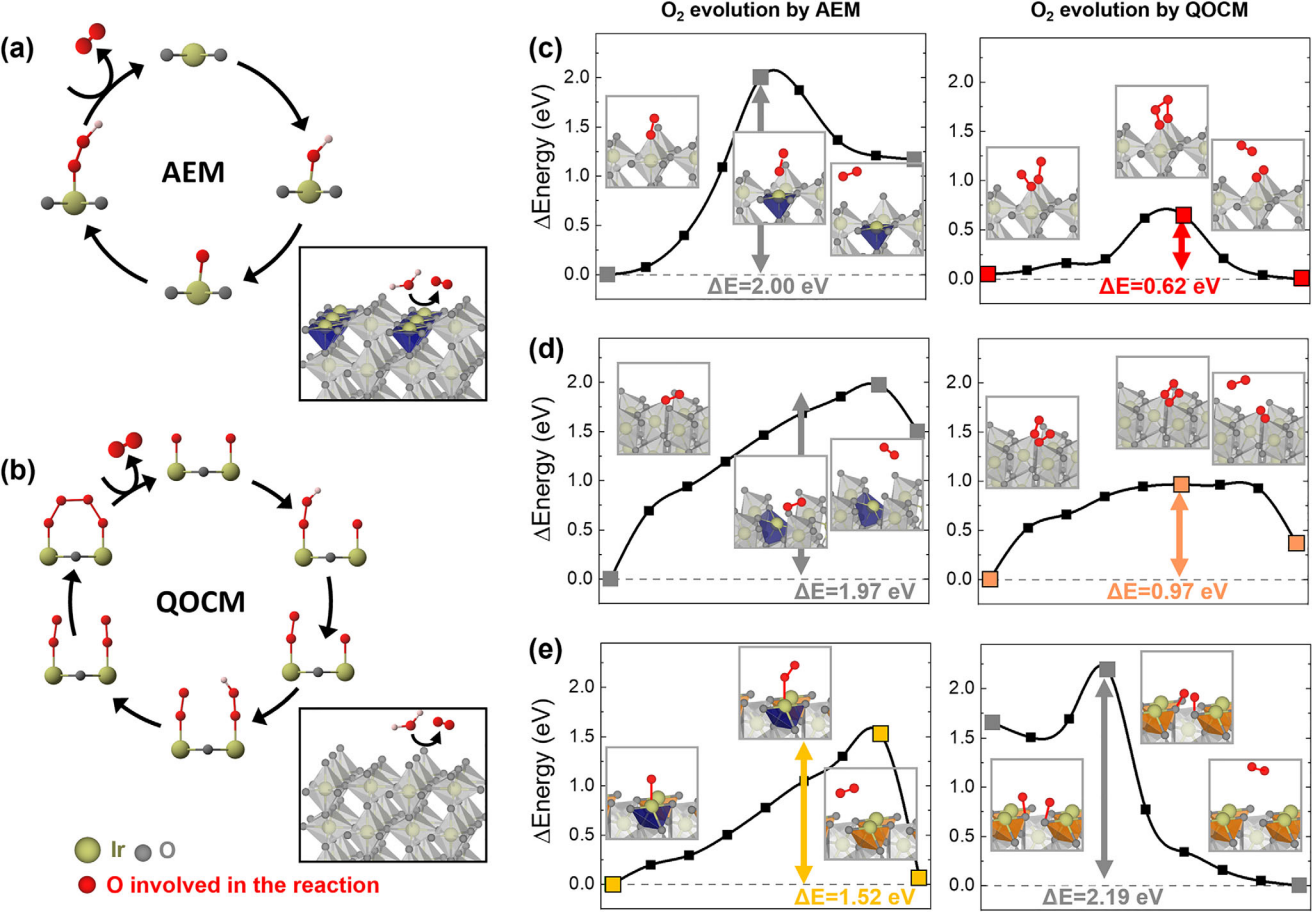
OER pathways and reaction energetics on IrO_2_ surfaces. (a,b) Schematics of the adsorbate evolution mechanism (AEM) and quadruple oxygen coupling mechanism (QOCM) for OER on IrO_2_. The highlighted red oxygen atoms indicate the oxygen species participating in the reaction. Insets show the corresponding local surface coordination environments. (c–e) Reaction energy diagrams for O_2_ evolution on IrO_2_ (110), (101), and (001), respectively, for the AEM (left) and QOCM (right) pathways. Insets show the initial, intermediate, and final states of each pathway. The horizontal dashed lines mark the lowest‐energy states, and ΔE denotes the energy barrier for the key O_2_ formation step.

To assess the thermodynamic relevance of the surface configurations under OER conditions, we evaluated their potential‐dependent surface coverages using the computational hydrogen electrode approach (Figure S11). Oxygen‐rich configurations on the (110) and (101) surfaces become favorable above ∼1.38 V vs RHE, whereas O adsorption on the undercoordinated Ir site of the (001) surface becomes favorable only above ∼1.53 V, supporting the configurations used in the subsequent reaction‐pathway calculations. Free‐energy analysis further identified the OH + OOH → OH + OO step as the potential‐determining step for the QOCM on both the (110) and (101) surfaces, with theoretical overpotentials of 0.279 and 0.345 V, respectively (Figure S12).

To examine the kinetics of oxygen coupling, we calculated O─O bond formation barriers using the climbing‐image nudged elastic band (cNEB) method. The corresponding barriers are 0.52 eV for the (110) surface and 0.75 eV for the (101) surface, whereas a substantially higher barrier of 1.62 eV is obtained for the (001) surface (Figure S13). These results indicate that O─O bond formation is kinetically more accessible on the (110) and (101) surfaces, particularly on the (110) surface.

We further evaluate the O_2_‐evolution barriers for the AEM and QOCM. For the (110) surface, O_2_ evolution through the Ir–OOOO–Ir state requires a barrier of 0.62 eV, substantially lower than the 2.00 eV barrier for direct O_2_ desorption, demonstrating a clear preference for the QOCM. The (101) surface shows the same trend, with a lower barrier for QOCM than for the AEM, 0.97 versus 1.97 eV. In contrast, the Ir–OOOO–Ir configuration is unstable on the (001) surface, and O_2_ evolution instead proceeds through the AEM pathway with a barrier of 1.52 eV. Overall, these thermodynamic and kinetic results indicate a clear energetic preference for the QOCM on the experimentally identified (110) and (101) surface structures, whereas the (001) surface is more consistent with an Ir‐centered AEM‐like pathway. The present mechanistic interpretation is therefore based on the calculated energetic preference of the experimentally identified surface structures, while direct experimental discrimination between AEM and QOCM remains an important challenge, as discussed in Text S1.

### 2.4 Electrochemical Measurement

To experimentally test this mechanistic prediction, we measured and compared the OER activities of IrO_2_ films with three distinct crystal orientations. Figure 5a shows the variation of OER current density under a cyclic anodic potential applied to the film, ranging from 1.0 to 1.65 V relative to the reversible hydrogen electrode (RHE). A clear dependence of OER activity on crystal orientation is observed. The current density follows the order (110) > (101) > (001), consistent with the calculated activation‐barrier sequence, as quantitatively analyzed in Figure 5b.

**FIGURE 5:**
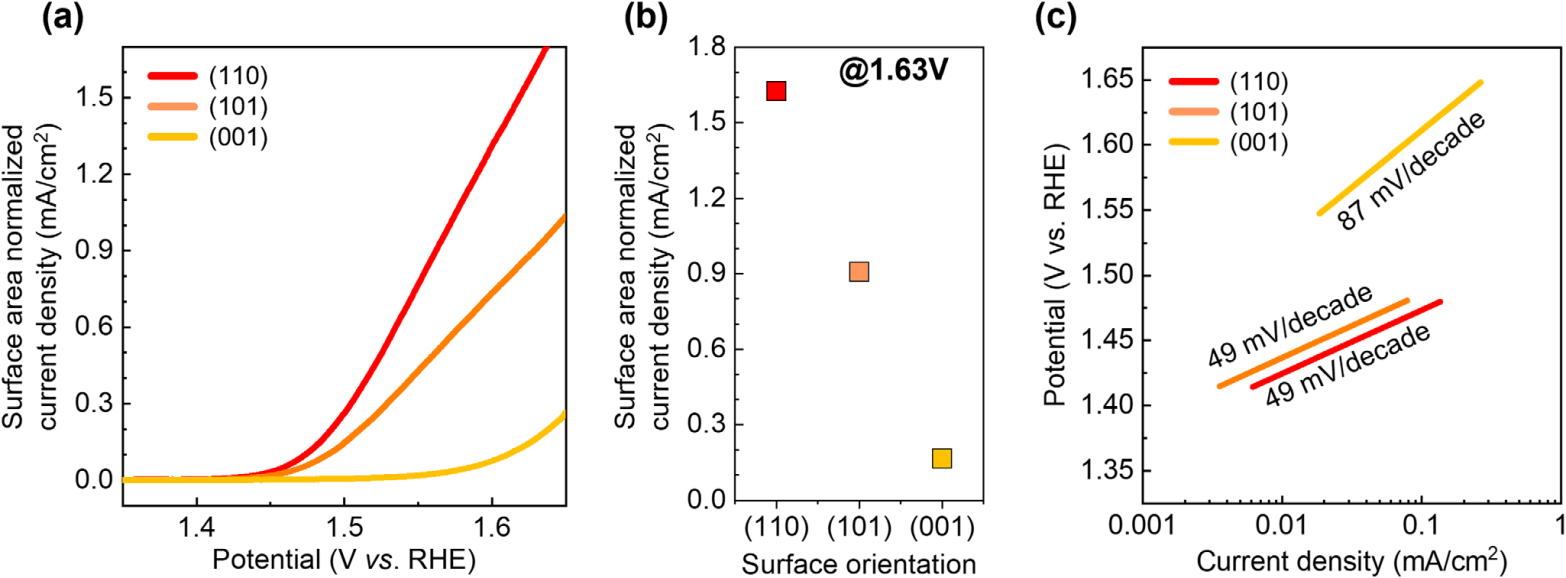
Surface orientation‐dependent oxygen evolution activity in IrO_2_ films. (a) Comparison of OER current densities depending on surface orientation. The current densities were normalized by the AFM‐derived 3D surface area. (b) Current density extracted from the cyclic voltammetry (CV) curves (1.63 V). (c) Comparison of the Tafel slopes obtained from the first CV cycle for different surface orientations.

To gain further insight into the OER characteristics, we examined the Tafel slopes for each surface orientation as shown in Figure 5c. Despite variations in the absolute Tafel slope values during cycling, the orientation‐dependent trend is maintained (Figure S14). The similar Tafel slopes of the (110) and (101) surfaces are consistent with similar apparent OER kinetics, whereas the distinct Tafel slope of the (001) surface suggests different kinetic behavior [36, 37, 38, 39]. These electrochemical trends are consistent with the computationally predicted mechanistic distinction, in which the (110) and (101) surfaces favor the oxygen‐centered QOCM, whereas the (001) surface is more consistent with an Ir‐centered AEM.

## 3 Conclusion

In this study, we demonstrated that the OER activity and reaction pathway of rutile IrO_2_ are critically governed by crystallographic surface orientation and the associated surface coordination environment. By combining atomic‐resolution STEM imaging with electronic‐structure and reaction‐barrier calculations, we directly identified the surface terminations of IrO_2_ and established a correlation between surface coordination, electronic structure, and catalytic mechanism. The (110) and (101) surfaces, dominated by fully coordinated IrO_6_ motifs, exhibit enhanced oxygen‐related electronic activity and preferentially favor the oxygen‐centered QOCM pathway with low activation barriers. In contrast, the (001) surface, characterized by undercoordinated IrO_4_ motifs, favors an Ir‐centered AEM pathway with a substantially higher activation barrier. Electrochemical measurements consistently confirmed the orientation‐dependent activity trend of (110) > (101) > (001).

Our results more broadly identify surface coordination as a key descriptor governing oxygen participation and reaction‐pathway selection in rutile oxide electrocatalysts. These findings provide direct experimental insight into the atomic‐scale origin of orientation‐dependent OER activity and establish a framework for designing high‐performance oxide catalysts through surface‐coordination and electronic‐structure engineering.

## 4 Experimental Section

### 4.1 Sample Growth, Fabrication, and Characterization

Epitaxial IrO_2_ was grown on TiO_2_ (110), (101) and (001) substrates by RF magnetron sputtering. The IrO_2_ films were grown at 400°C at a pressure of 30 mTorr with 10% oxygen partial pressure. The target power was 20 W. After growth, the sample was cooled in an O_2_ atmosphere. The sample surfaces were observed by atomic force microscopy. X‐ray characterization of the samples was performed at room temperature by using a four‐circle X‐ray diffractometer equipped with Cu‐Kα_1_ radiation.

### 4.2 Electrochemical and Electrical Tests and Measurements

All electrochemical reactions and measurements were conducted with a potentiostat (Biologic SP‐300) in a 0.1 M HClO_4_ aqueous solution (pH 1). A Pt counter electrode and a saturated Ag/AgCl/NaCl reference electrode were used. The measured potential values vs. the Ag/AgCl/NaCl reference electrode were converted into the RHE scale. For thin‐film samples, the substrate and the connecting copper wire were completely covered with chemically inert insulating epoxy resin (Henkel Loctite EA 9460) after application of silver paint (Cans Elcoat P‐100) on the back side of a thin‐film sample so as to expose the film surface only. The measured current was first normalized by the exposed geometric area of each electrode. To account for differences in surface roughness, the geometric‐area‐normalized current density was subsequently divided by the AFM‐derived roughness factor, defined as the ratio of the actual 3D surface area to the projected area, yielding the current density normalized to the actual 3D surface area. All electrolyte solutions were presaturated by bubbling O_2_ for 30 min under constant O_2_ bubbling. Cyclic potential was applied to the samples at a rate of 10 mV/s, sweeping from 1.0 to 1.65 V vs. RHE to record the first‐cycle OER current density in each case. Samples were taken out of the electrolyte solution to remove O_2_ gas bubbles adsorbed on the film surface every five anodic cycles. Electrochemical impedance spectroscopy was also carried out in the same potentiostat in a frequency range from 0.1 Hz to 1 MHz with an amplitude of 10 mV for iR correction of the uncompensated series resistance.

### 4.3 Scanning Transmission Electron Microscopy

The crystal structures of IrO_2_ films were analyzed using STEM (JEM‐ARM200F, JEOL) at 200 kV equipped with the fifth‐order spherical aberration corrector (ASCOR, CEOS GmbH) at the Materials Imaging and Analysis Center, Pohang University of Science and Technology (POSTECH),  Republic of Korea (NFEC‐2018‐01‐241864). The optimum size of the electron probe was ∼0.7 Å. The collection semi‐angle of the HAADF detector ranged from 45 to 180 mrad for clear Z‐contrast images. Annular bright‐field (ABF) images were acquired with a 10–20 mrad inner‐outer collection semi‐angle, and bright‐field (BF) images were acquired with a 10 mrad for light elements. Although ABF‐STEM is widely used to visualize oxygen structures [29, 40, 41], the significant atomic number difference between Ir and O makes it challenging to accurately observe the oxygen structure with conventional ABF‐STEM imaging, as shown in the STEM simulation images (Figure S15). Therefore, enhanced ABF‐STEM (eABF‐STEM) imaging was employed to improve the visibility of oxygen columns near the surface and to support the identification of surface terminations. eABF‐STEM is constructed by taking the difference between ABF and BF signals collected from different regions of the bright‐field disk, thereby enhancing the detectability and signal‐to‐noise ratio of light‐element columns [40 ^,^ 41]. In this study, eABF‐STEM was used to support the identification of oxygen‐containing surface terminations in the IrO_2_ films.

Surface‐to‐bulk Ir displacement was analyzed using HAADF‐STEM intensity maps to evaluate whether the surface Ir framework deviates from the underlying bulk lattice. The bulk Ir reference positions were defined from the underlying lattice region, and the apparent positions of the surface Ir columns were compared with the corresponding projected bulk‐derived reference positions, as illustrated in Figure S16. The analysis revealed only negligible deviations from the bulk lattice positions, indicating that the surface Ir framework remains largely bulk‐like without clear evidence of pronounced surface reconstruction.

Multiple STEM image frames were acquired and subsequently aligned using Smart Align with rigid registration to correct for specimen drift and scan distortions. The raw STEM images were filtered to reduce background noise using Difference Filter (Filters Pro, HREM Research Inc., Japan). STEM samples were prepared using a dual‐beam focused ion beam (FIB) system (Nanolab G3 CX, FEI). We used a Ga‐ion beam with an acceleration voltage of 30 kV and then used lower acceleration voltages from 5 to 2 kV. Prior to FIB sampling, a protective layer of 60 nm carbon was deposited on the sample surface using a carbon evaporation coater (EM ACE200, Leica) at the Materials Imaging & Analysis Center of POSTECH, Republic of Korea (NFEC‐2021‐10‐273451).

### 4.4 Density Functional Theory Calculations

Density functional theory (DFT) calculations were performed using the projector augmented‐wave method [42], which was implemented in the Vienna Ab initio Simulation Package (VASP) code [43]. The exchange–correlation functional was constructed using the generalized gradient approximation (GGA) in the Perdew–Burke–Ernzerhof (PBE) scheme [44]. Electronic wave functions have been demonstrated using a plane‐wave basis set with an energy cutoff of 500 eV. Convergence criteria for energy and force were set at 10^−8^ eV and 0.005 eV Å^−1^ for IrO_2_ bulk structures. A Γ‐centered 15 × 15 × 20 k‐point grid was employed to integrate the Brillouin zones for IrO_2_ bulk structures. For IrO_2_ slab calculations, 5 layers of IrO_2_ oriented along the (110), (001) and 6 layers of IrO_2_ oriented along the (101) direction were considered with a vacuum layer of 20 Å, which are sufficiently thick to avoid interaction. Atomic positions of the slab were relaxed, keeping the inner IrO_2_ layer fixed with convergence criteria of 10^−5^ eV and 0.02 eV Å^−1^ for energy and force, respectively. To compare activation energy barriers among different orientations, the climbing image nudged elastic band (cNEB) method [45] was employed with convergence criteria of 10^−5^ eV for energy and 0.05 eV Å^−1^ for force.

## Author Contributions

**Gahee Noh**: conceptualization, writing – original draft, formal analysis, investigation, visualization. **Seong‐Sik Kim**: writing – review and editing, investigation, visualization. **Hyungwoo Lee**: formal analysis, methodology, validation. **Jaejin Hwang**: methodology, validation, formal analysis. **Michael Patton**: investigation, formal analysis, resources. **Owen Ericksen**: investigation, formal analysis, resources. **Tae Gyu Yun**: investigation. **Seung‐Hyun Heo**: investigation. **Suho Lee**: investigation. **Yurim Hong**: investigation, validation. **In Su Lee**: supervision, writing – review and editing, resources. **Daesu Lee**: supervision, resources. **Chang‐Beom Eom**: supervision, resources. **Jaekwang Lee**: supervision, resources. **Minseok Choi**: supervision, resources. **Sung‐Yoon Chung**: supervision, resources. **Si‐Young Choi**: supervision, funding acquisition, project administration, writing – review and editing.

## Conflicts of Interest

The authors declare no conflicts of interest.

## Supporting information




**Supporting File**: advs78589‐sup‐0001‐SuppMat.pdf.

## Data Availability

The data that support the findings of this study are available from the corresponding author upon reasonable request.
