# Supplementary material for: Surface Coordination‐Dependent Oxygen Evolution Pathways in Rutile IrO2
Source: Adv Sci (Weinh). 2026 Nov 5:e78589. Online ahead of print. doi: 10.1002/advs.78589 (PMC13735428; doi:10.1002/advs.78589)
Supplement: Supplementary file 1 [file ADVS-9999-e78589-s001.pdf]

Surface Coordination-Dependent Oxygen Evolution Pathways in Rutile IrO_2

Supporting information

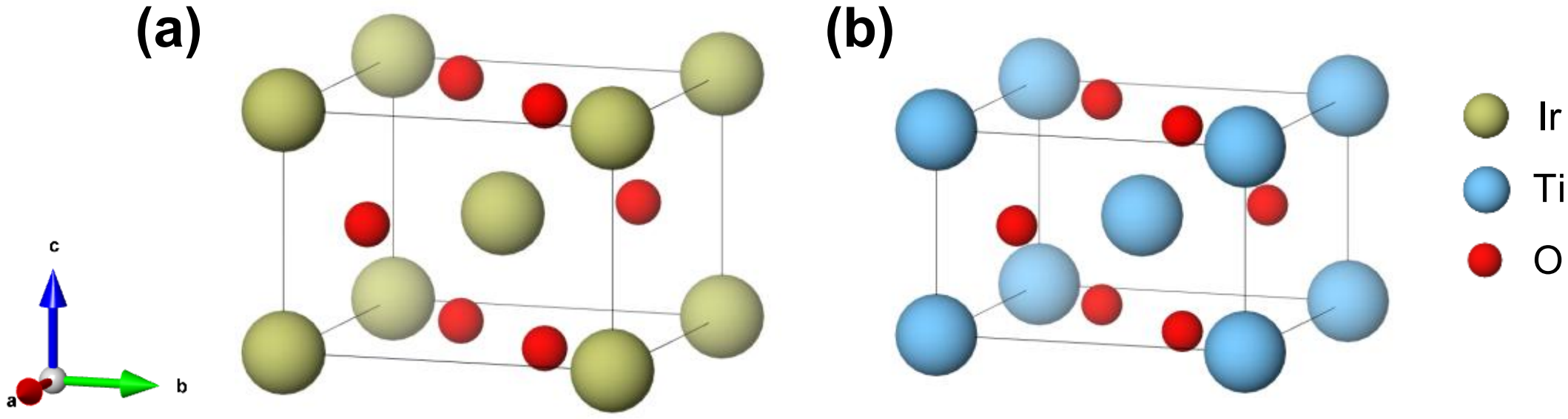

Figure S1. Crystal structure of (a) IrO_2 and (b) TiO_2 rutile structure.

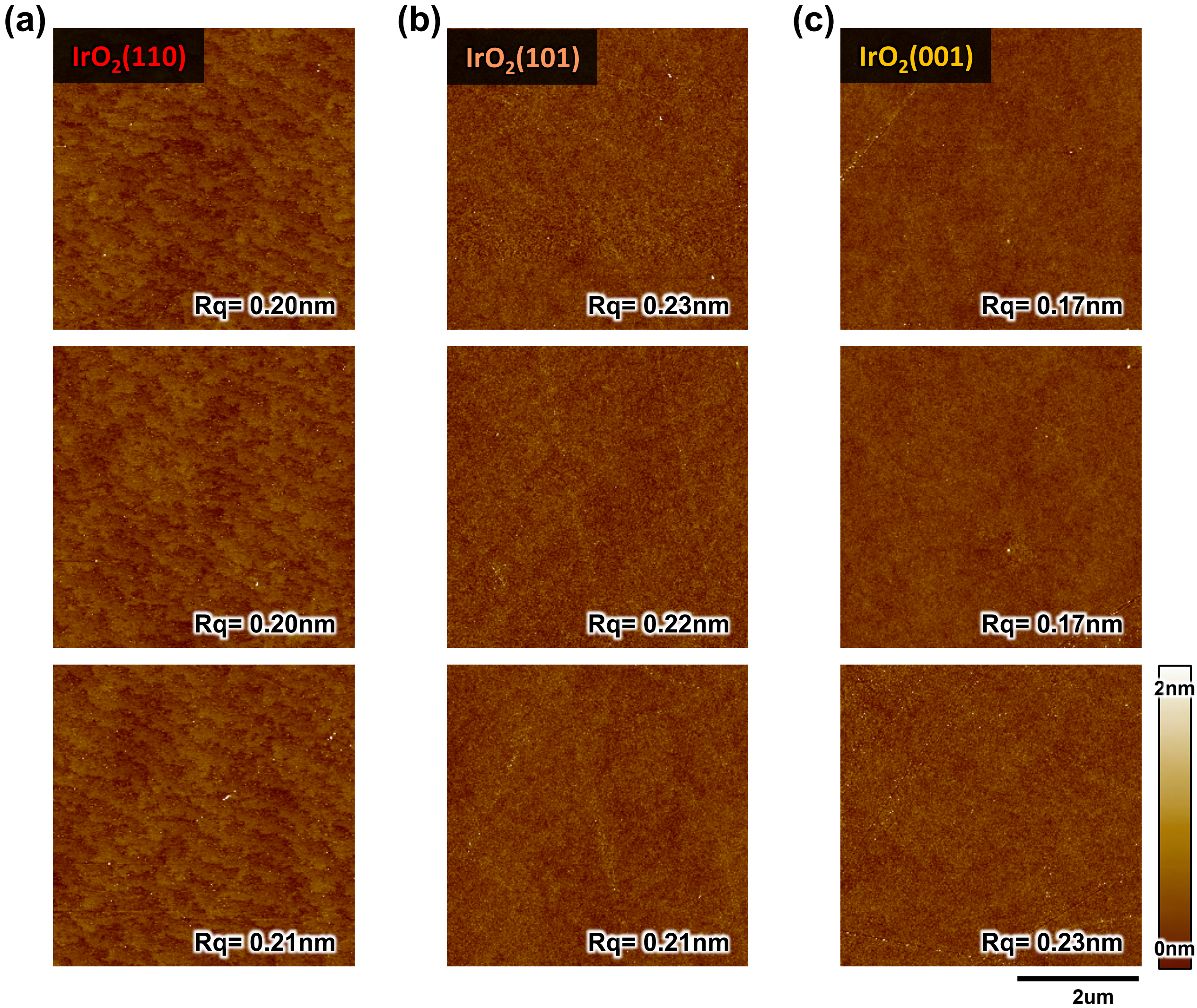

Figure S2. AFM images of epitaxial IrO_2 films measured at multiple spatially separated regions. Representative 5 x 5 μm^2 AFM images acquired from three different regions of the IrO_2 (a) (110), (b) (101), and (c) (001). The corresponding root-mean square surface roughness (Rq) values are indicated in each image, showing consistently low surface roughness across different regions of three orientations.

	(110)	(101)	(001)
RMS and Surface area of region 1	0.20 nm / 25.0067 μm^2	0.23 nm / 25.0713 μm^2	0.17 nm / 25.0067 μm^2
RMS and Surface area of region 2	0.20 nm / 25.0075 μm^2	0.22 nm / 25.0125 μm^2	0.17 nm / 25.0072 μm^2
RMS and Surface area of region 3	0.21 nm / 25.0064 μm^2	0.21 nm / 25.0159 μm^2	0. 23 nm /25.0163 μm^2
Mean 3D surface area	**25.0068** μm^2	**25.0333** μm^2	**25.0100** μm^2
Standard deviation	0.0005	0.0330	0.0054
Roughness factor	**1.0003**	**1.0013**	**1.0004**

Table S1. AFM-derived 3D surface area of each IrO_2 orientation. The roughness factor was calculated as the ratio of the actual 3D surface area obtained from AFM to the projected scan area (25 μm^2).

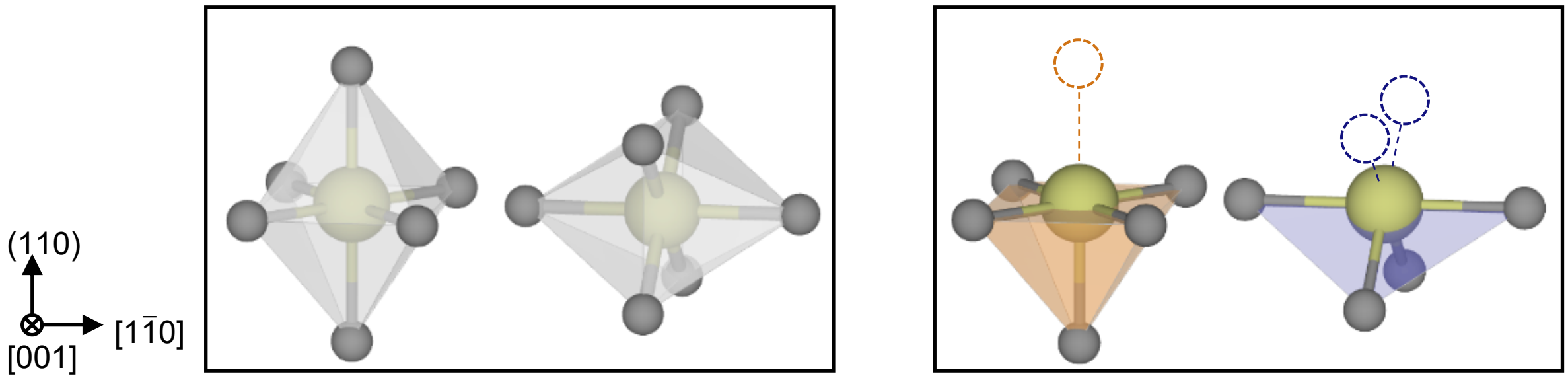

Figure S3. bulk-like IrO_6 structure (left) and IrO_5 and IrO_4 structure (right)

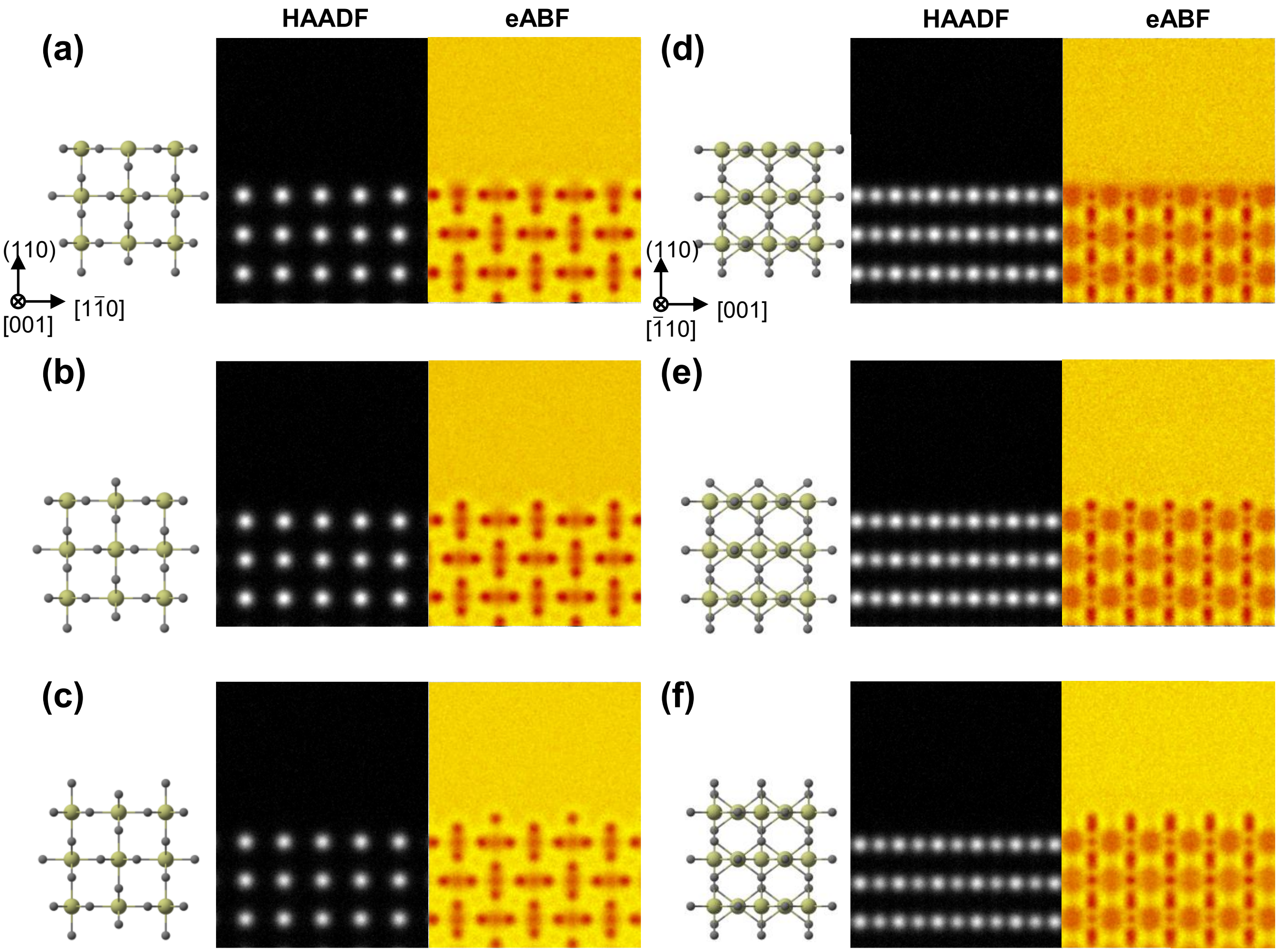

Figure S4. STEM simulation images of IrO_2 (110) surface structure. (a)-(c) Surface structure candidates of IrO_2(110) surface with [001] projection. (d)-(f) Surface structure candidates of IrO_2(110) surface with [$\bar{1}$10] projection. The differences among the three structures are not discernible in the HAADF-STEM simulation images; however, they are clearly observed in the eABF images.

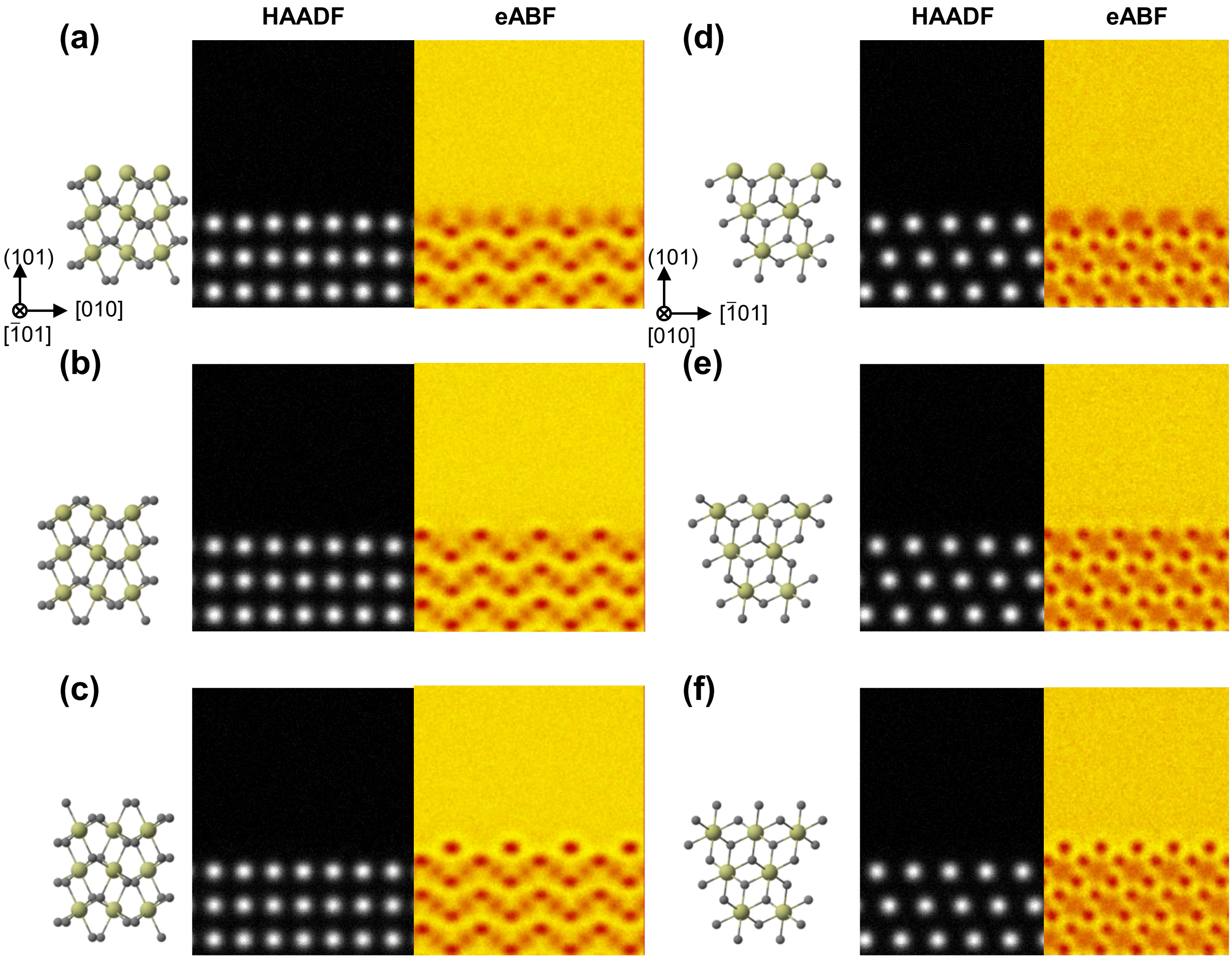

Figure S5. STEM simulation images of IrO_2 (101) surface structure. (a)-(c) Surface structure candidates of IrO_2 (101) surface with [$\bar{1}$01] projection. (d)-(f) Surface structure candidates of IrO_2 (101) surface with [010] projection. The differences among the three structures are not discernible in the HAADF-STEM simulation images; however, they are clearly observed in the eABF images.

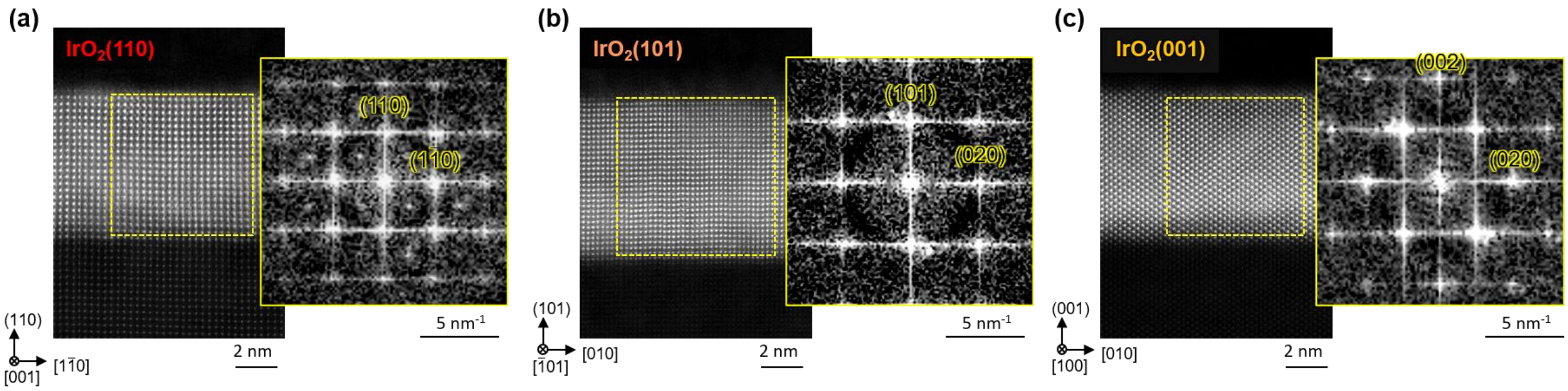

Figure S6. Distinct FFT patterns of IrO_2 (a) (110), (b) (101), and (c) (001).

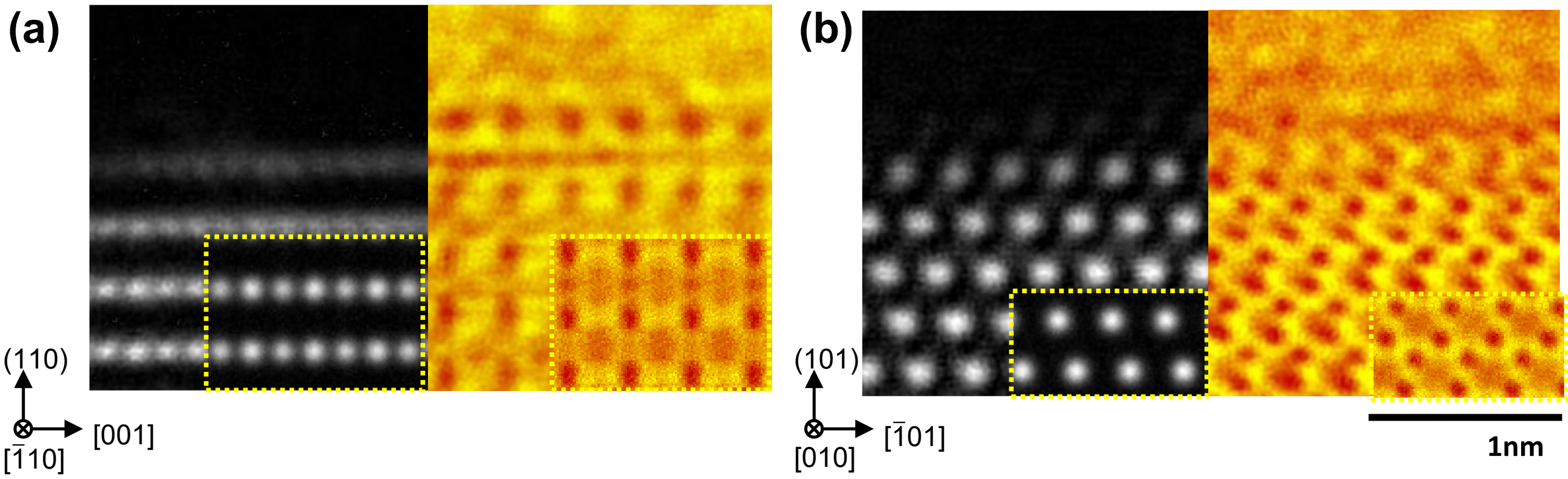

Figure S7. Direct observation of IrO_2 (110) and (101). (a) and (b) High-magnification HAADF-STEM (left) and eABF-STEM (right) images. To precisely observe each surface structure, the projections were also analyzed after in-plane 90-degree rotation. The yellow dashed boxes indicate HAADF and eABF simulation images of IrO_2.

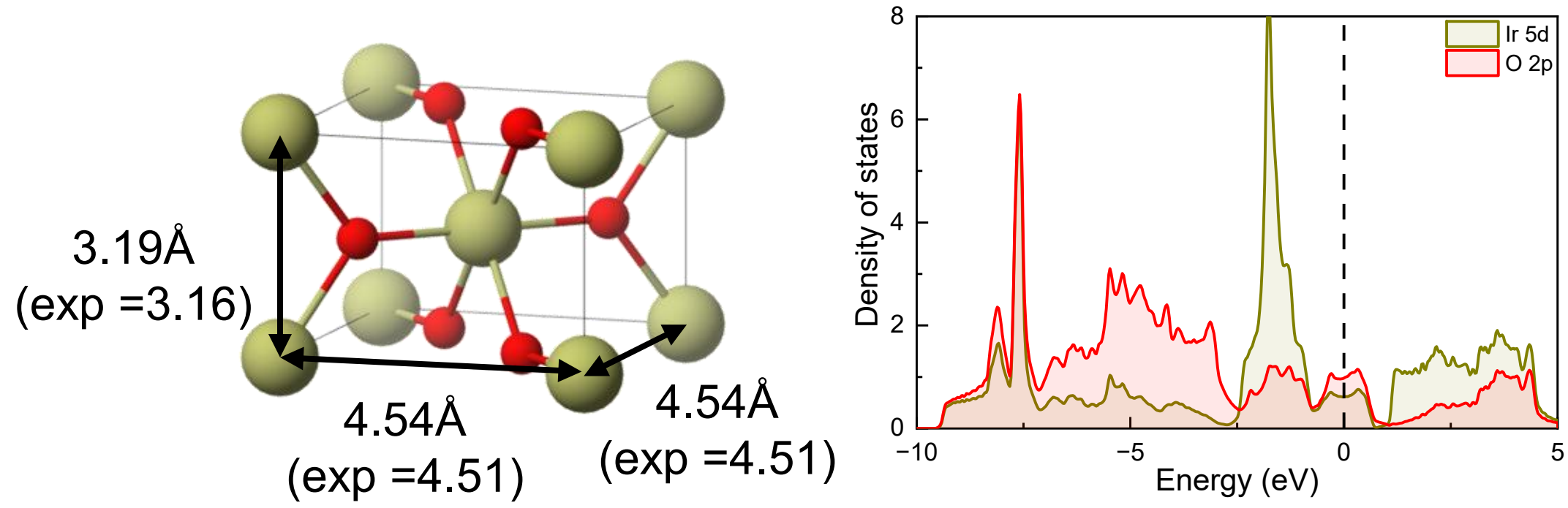

Figure S8. Density of states calculation of bulk IrO_2.

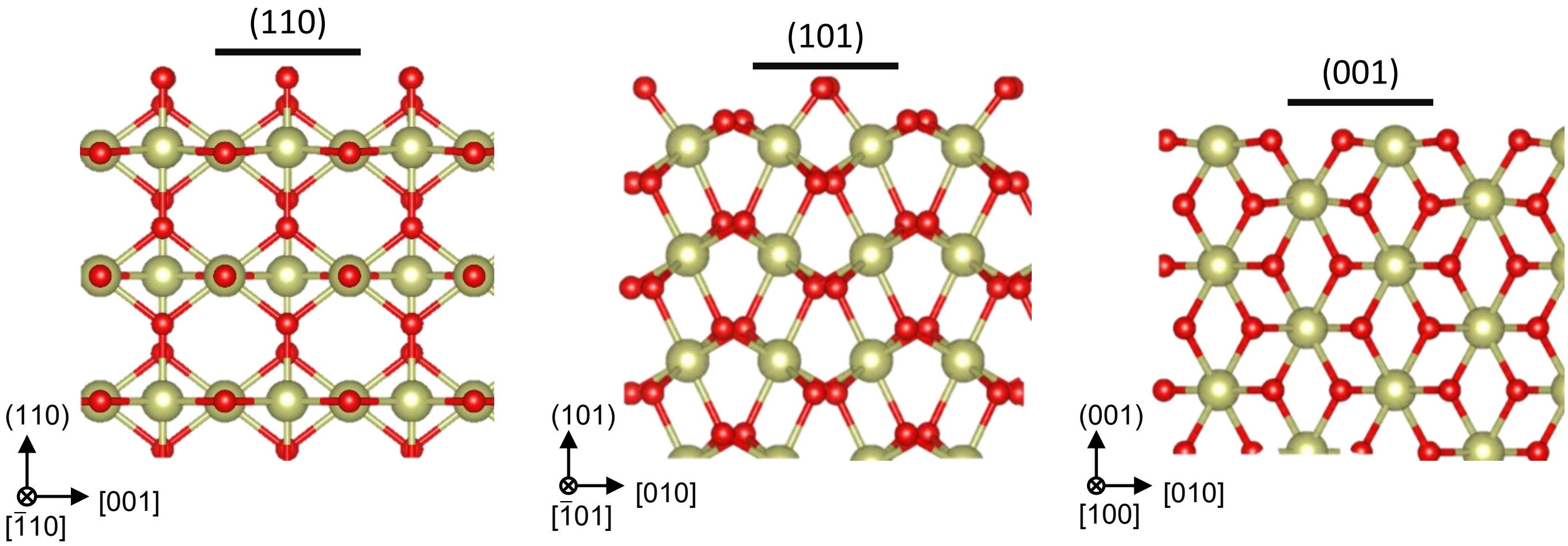

Figure S9. Calculated surface structure based on STEM experiment with minor reconstruction.

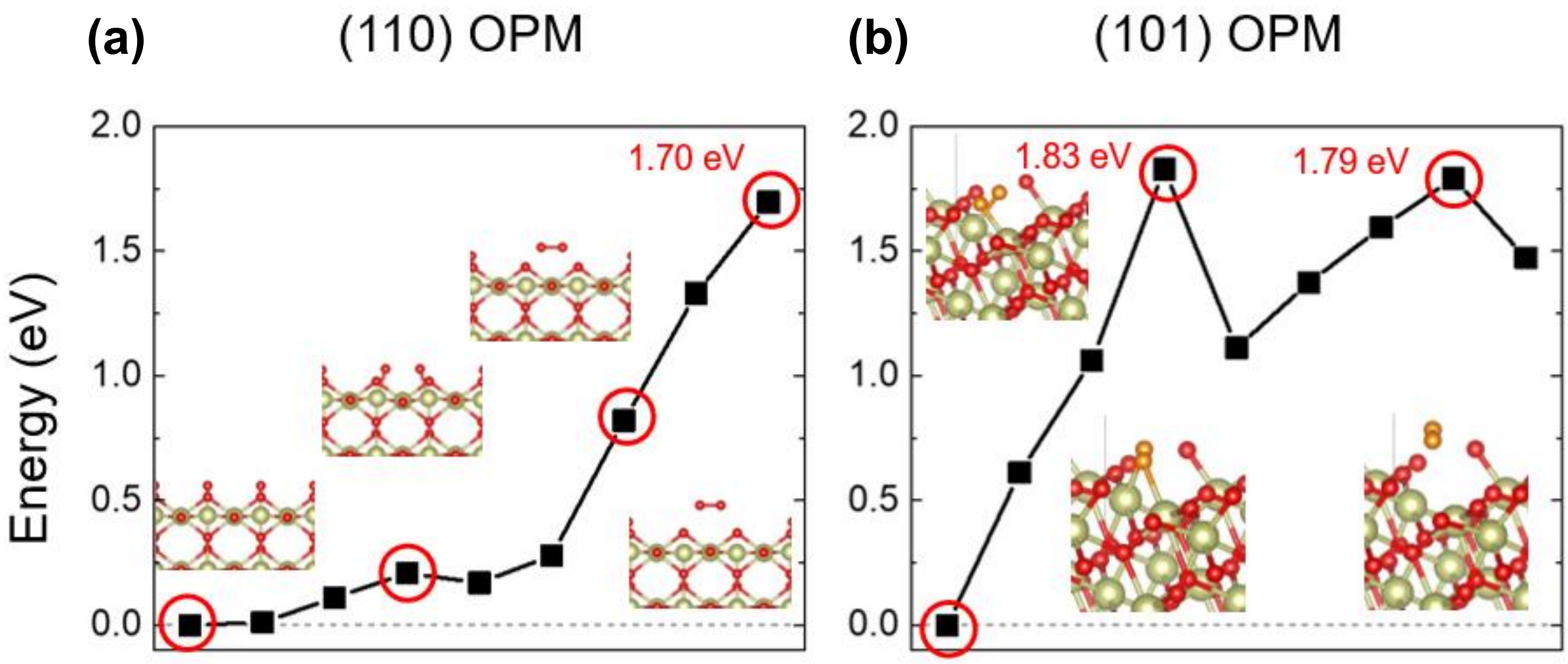

Figure S10. The calculated activation barriers along the OPM pathway for the (a) (110) and (b) (101) surfaces.

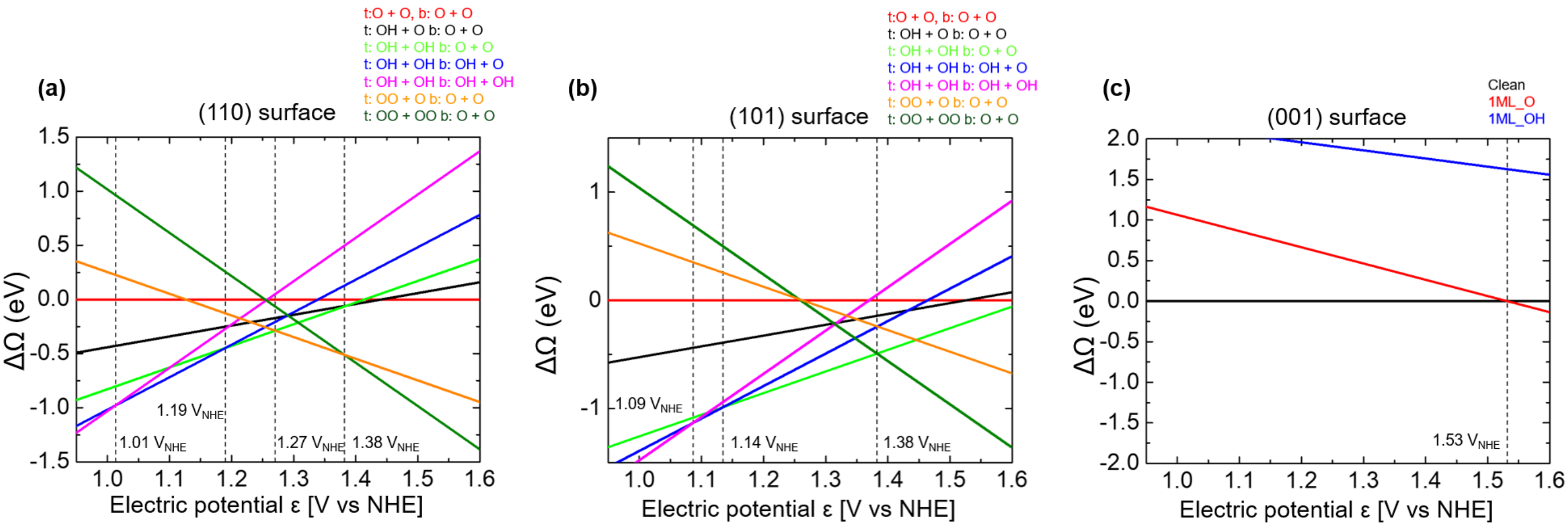

Figure S11. The calculated potential-dependent surface coverage diagrams of IrO_2 (a) (110), (b) (101), and (c) (001) surface. Oxygen-rich configurations on the (110) and (101) surfaces become favorable above ~1.38 V vs RHE, whereas O adsorption on the undercoordinated Ir site of the (001) surface becomes favorable only above ~1.53 V.

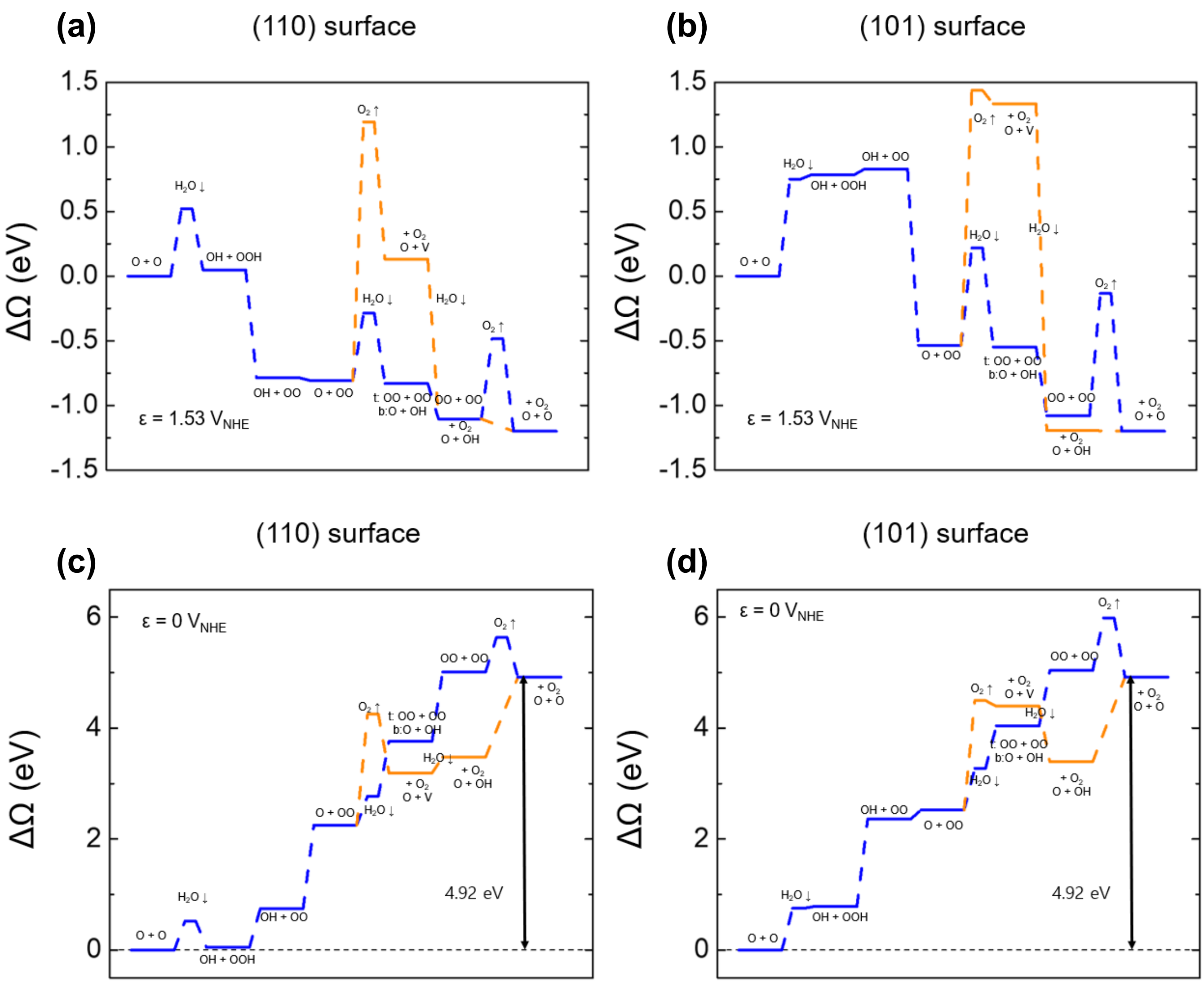

Figure S12. Free-energy diagrams for the AEM and QOCM pathways on the IrO_2 surfaces. (a) (110) and (b) (101) surfaces at 1.53 V vs NHE. (c) (110) and (d) (101) surfaces at 0 V vs NHE.

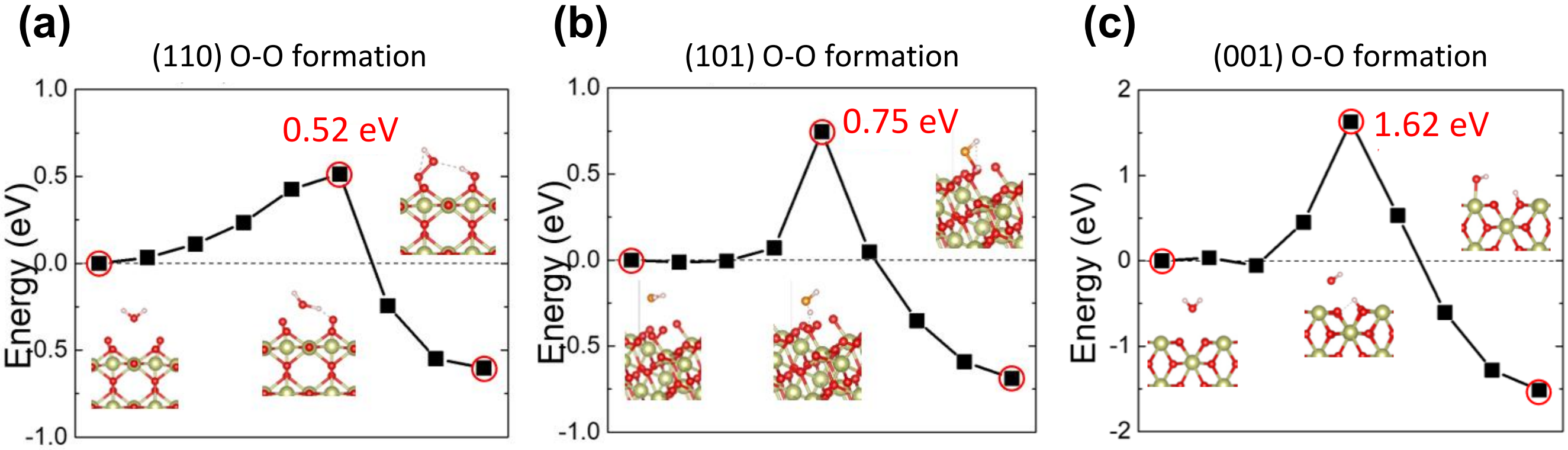

Figure S13. cNEB-calculated O–O bond-formation energy profiles for the QOCM pathway on IrO_2 surfaces. (a) (110), (b) (101), and (c) (001).

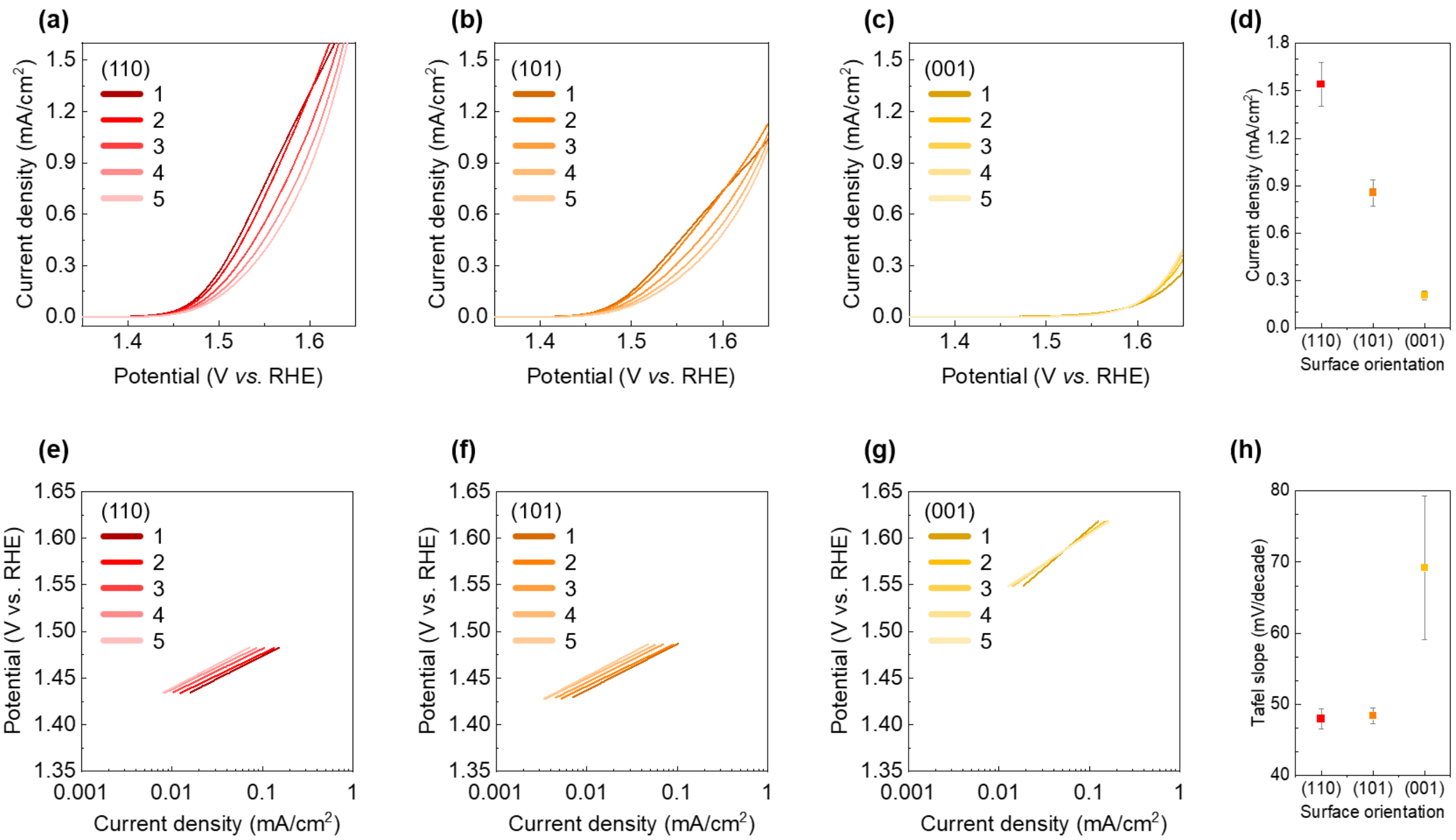

Figure S14. Cycle-dependent OER activity of epitaxial IrO_2 films. (a-c) CV curves of IrO_2 (110), (101), and (001) over five consecutive cycles. (d) Current densities at 1.63 V averaged over the five cycles. (e-g) Tafel slopes of IrO_2 (110), (101), and (001) over five consecutive cycles. (h) Tafel slopes obtained from the five consecutive cycles. Error bars in (d) and (h) represent the standard deviation over the five cycles.

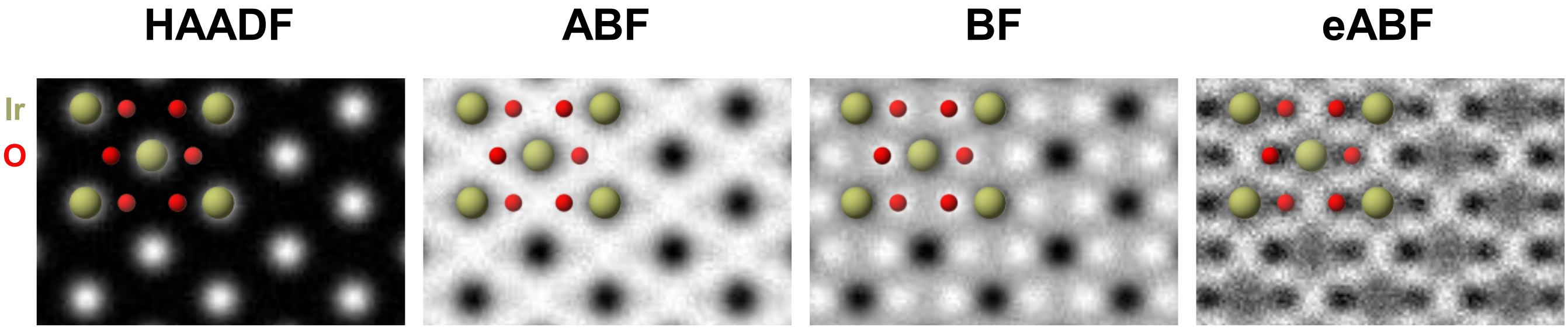

Figure S15. STEM simulation images of IrO_2 crystal structure.

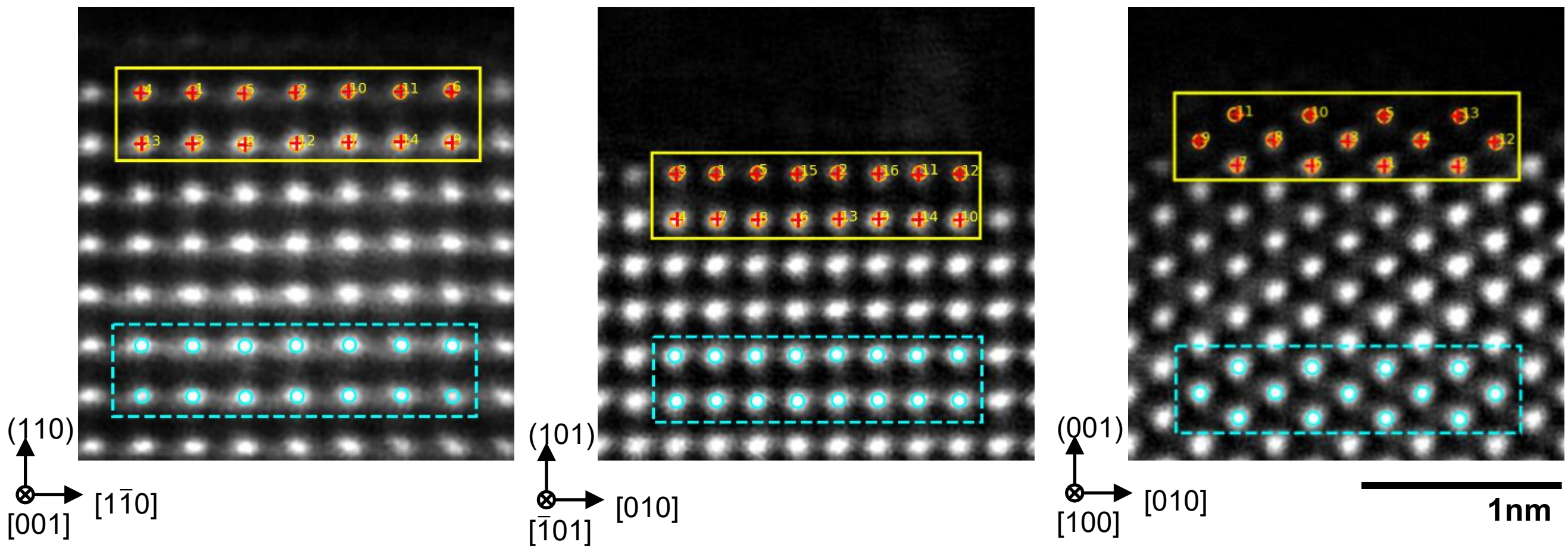

Figure S16. Surface-to-bulk Ir displacement analysis. Blue-boxed regions indicate the bulk Ir reference regions, and yellow-boxed regions indicate the surface Ir regions in the HAADF-STEM image. The bulk Ir reference positions were defined from the underlying lattice and compared with the apparent surface Ir column positions. This analysis was used to evaluate whether the surface Ir framework deviates from the bulk-like lattice structure.

Supporting text 1. Experimental distinction between AEM and QOCM

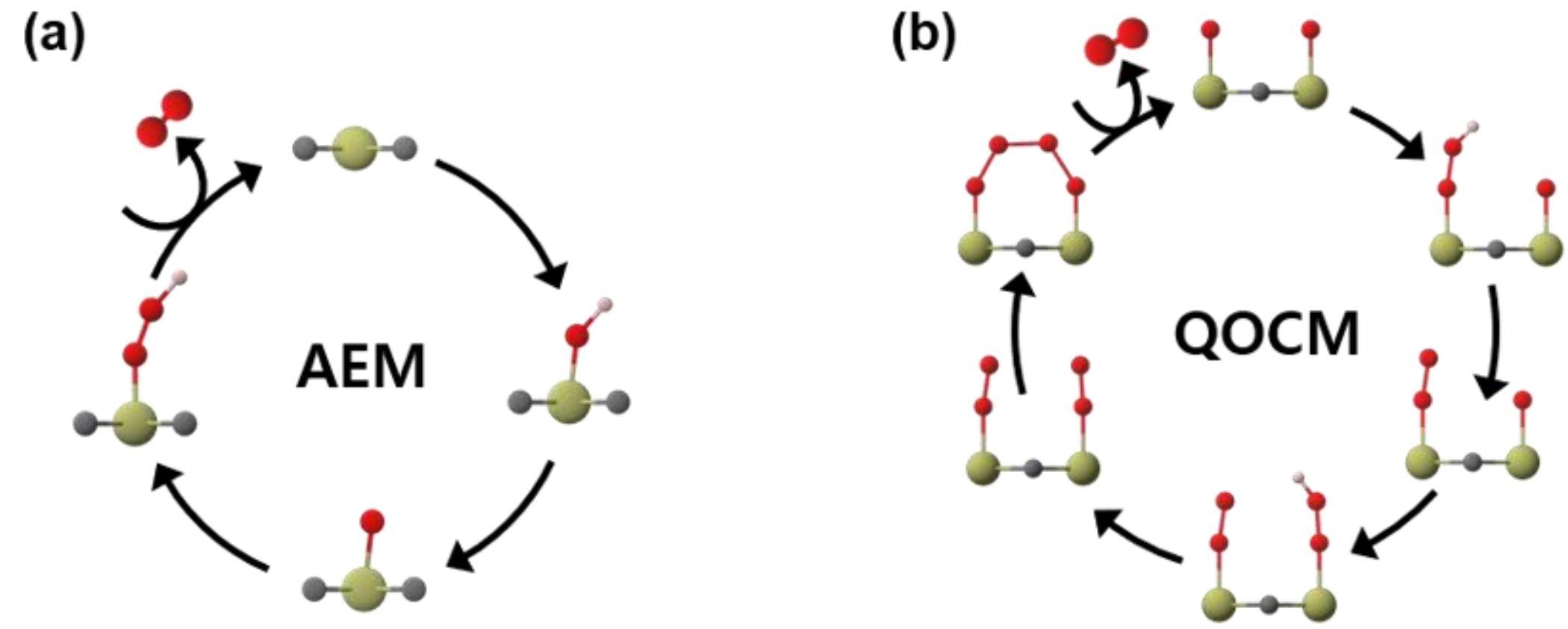

Figure S17. Schematics of OER pathways of (a) AEM and (b) QOCM. The two mechanisms share several intermediates but differ in the O-O coupling step and the required active-site configuration.

Direct experimental discrimination between the AEM and QOCM remains challenging because the two pathways share several adsorbed oxygen intermediates and mainly differ in the O–O coupling step. Isotope-labeling and chemical-probe approaches are particularly effective for identifying lattice-oxygen participation but do not provide a unique signature for distinguishing AEM from QOCM. Likewise, operando vibrational spectroscopy can probe surface oxygen species, but direct observation of the transient Ir–OOOO–Ir configuration proposed for QOCM is difficult because of its low steady-state population under OER conditions[1]. Accordingly, the mechanistic interpretation in this work is based on the relative thermodynamic and kinetic favorability of competing pathways calculated for the experimentally identified surface structures, while direct experimental discrimination between AEM and QOCM remains an important challenge.

[1] T. Binninger, M.-L. Doublet, *Energy Environ. Sci.* 2022, 15 (6), 2519–2528.
